# Response to immune-based augmentation treatment for depression: a potential role of immunosenescence

**DOI:** 10.1016/j.nsa.2026.106986

**Published:** 2026-02-06

**Authors:** Evelien Van Assche, Christa Hohoff, Sophia M. Wissing, Lea Steinbach, European College of Neuropsychopharmacology (ECNP) Pharmacogenomics & Transcriptomics Network, Bernhard T. Baune

**Affiliations:** a University of Münster, Münster, Germany; b Department of Psychiatry, Melbourne Medical School, The University of Melbourne, Melbourne, Australia; c The Florey Institute of Neuroscience and Mental Health, The University of Melbourne, Parkville, VIC, Australia

**Keywords:** Immunosenescence, Major depressive disorder, Celecoxib, Augmentation, Immune system, Antidepressant, Immunomethylomics

## Abstract

Immunological processes are increasingly in focus as factors contributing to major depressive disorder (MDD). Especially subtypes such as immunometabolic depression phenotypes have been linked to low-grade inflammation. Augmentation with immune-based therapies, e.g., celecoxib, is being tested for its efficacy in treating depression. Many physiological processes during life are also linked to immunological changes, particularly aging. As results from current trials with celecoxib augmentation remain inconclusive, we tested the hypothesis that age affects treatment efficacy. In total, 113 individuals with a diagnosis of major depressive disorder (*M*_age_ = 44, 56% women, *M*_MADRS_ = 27.7) had biomarkers available and were included in our analyses. Patients were recruited as part of a randomized controlled trial (RCT) and stratified by hsCRP (>3 mg/L or < 3 mg/L). All patients were treated with vortioxetine and randomized to receive either celecoxib (*N* = 55) or placebo (*N* = 58) by a randomized design. Patients were treated for 6 weeks (*M*_MADRS 6W_ = 20.2). We tested one main hypothesis: that age affects the treatment outcome of celecoxib as an immunomodulatory agent. To further explore the meaning of our results, we used epigenome-wide DNA methylation data (Illumina Infinium MethylationEPIC 850k BeadChip) available to estimate cell type compositions of neutrophils, monocytes, B-cells, CD4^+^ and CD8^+^ Lymphocytes, and natural killer cells (NK), using the Houseman method. The analyses were performed using linear regression and ANCOVA models, corrected for sex, hsCRP, years of education, BMI, and depression severity at baseline. Our analysis showed a statistically significant interaction between age and treatment condition on depression outcome (beta = −0.24; *p* = 0.045), with significant main effects for both variables in the model (intervention: beta = 10.88; *p* = 0.048, age: beta = 0.23; *p* = 0.01). Sex, hsCRP, and years of education were no statistically significant contributors. BMI was a marginally significant contributor in the model (beta = −0.25; *p* = 0.059). The intersection was identified at 45.5 years. Younger individuals treated with celecoxib showed a more pronounced reduction in MADRS than older individuals treated with celecoxib (*F*(1,52) = 5.74, *p* = 0.020). Further exploration showed that for individuals younger than 45 years, neutrophils at baseline might be associated with better treatment outcome (beta = 25.87; *p* = 0.051). For individuals older than 45 years, this was the case for B-cells, NK cells, and, suggestively, CD8^+^ cells (beta = −146.29; *p* = 0.014, beta = 129.39; *p* < 0.001, and beta = −20.23; *p* = 0.055, respectively). Our results indicate that response to celecoxib as an augmentation treatment for depression can be age-dependent, with younger patients responding better to treatment. In addition, immunological cell type profiles at baseline differ between both age groups, supporting the hypothesis that changes in treatment efficacy might follow from immunological changes that happen during aging. Our result fits with the growing body of literature focusing on immunosenescence and that aging of the immune system is relevant for treatment response and personalized treatment choices. Replication in an independent sample is needed to confirm the role of age in immune-focused treatment strategies for depression.

## 1 Introduction

Depression and other psychiatric disorders are increasingly being linked to immunological processes (Miller and Raison, 2016; Penninx et al., 2025). These processes affect a wide range of brain-related mechanisms, contributing to the emerging field of immuno-psychiatry (Miller and Raison, 2016; Ravi et al., 2021; Miller et al., 2025). Apart from inflammation per se, an impaired blood-brain barrier function has also been linked to immunological processes, as well as psychiatric disorders (Solak et al., 2025; Greene et al., 2020). Immunological processes are not only crucial for a healthy brain, but they also protect against external influences, such as infection, toxic substances, or cancerous processes (Zengeler and Lukens, 2021).

Within this context, medications that modify the immune system, such as corticosteroids or immunosuppressants, have been shown to induce affective symptoms, both manic or depressive, on multiple occasions (Baune et al., 2012). It has also been observed that many individuals with major depressive disorder may have systemic low-grade inflammation with sub-clinically increased C-reactive protein (CRP) levels (Penninx et al., 2025; Osimo et al., 2019). This observation has contributed to the concept of immunometabolic depression. Inflammation and metabolic alterations, such as obesity or altered glucose metabolism, are typical for this subtype of depression (Penninx et al., 2025; Milaneschi et al., 2019). It also shows signs of overall hypometabolism, with increased sleep, reduced energy, etc (Penninx et al., 2025; Milaneschi et al., 2019, 2020).

Based on these observations, clinical trials have assessed the potential value of anti-inflammatory agents as an augmentation strategy to antidepressant treatment. The main hypothesis has been that patients with major depression and a higher inflammatory load could benefit from an anti-inflammatory augmented treatment strategy, e.g., celecoxib or minocyclin (Penninx et al., 2025; Baune et al., 2021). However, results have been inconsistent (Baune et al., 2021; Wang et al., 2022), but more studies are planned (Zwiep et al., 2023; Wessa et al., 2024). The inconsistent results can be due to methodological aspects, for example, in the same trial, the beneficial effects of celecoxib were not seen at the end of the RCT, but only after a follow-up period of 35 weeks (Sampson et al., 2025). However, the inconclusive signals between studies can also be due to heterogeneity at the patient level, in terms of clinical manifestations or biography.

Heterogeneity is a heavily discussed feature of depression. Many factors contribute to the different presentations of depression in different individuals (Milaneschi et al., 2020). This includes biological sex, early life experiences, and age of onset (Szymkowicz et al., 2023; Gee, 2021). Aging, too, is a physiological process intertwined with immunological changes (Baune et al., 2012; Szymkowicz et al., 2023).

In addition, depression has been linked to immunological processes repeatedly in older individuals (Baune et al., 2012; Szymkowicz et al., 2023; Straka et al., 2021). Aging itself is a physiological process closely linked to immunological changes (Fulop et al., 2023). The concept of “immunosenescence” harbors the observation that aging affects the immune system per se (Lee et al., 2022; Liu et al., 2023). These changes in the immune system that come with aging have been linked to depression (Teixeira et al., 2025), including premature T-cell aging (Simon et al., 2023a), as well as other immune-related cell types (Gao et al., 2024). Furthermore, changes in the blood-brain-barrier permeability are a physiological process during healthy aging, but they can be worsened in neurodegenerative disorders, including depression (Knox et al., 2022). Hence, aging may modify antidepressant effects, too. For instance, a previous meta-analysis suggested that younger age was associated with a greater response to serotonergic than noradrenergic agents, which was attributed to the developmental process of the noradrenergic system (Ossola et al., 2025).

The overarching purpose of precision psychiatry is to tackle heterogeneity by identifying individuals and patient groups who benefit particularly from a specific intervention. Overall, the existing literature suggests that age and aging can be relevant for the clinical response to an immune-modulatory augmentation strategy for depression. For now, the results of available RCTs are inconsistent. Hence, we intend to investigate the role of age and aging in these heterogeneous observations regarding the efficacy of celecoxib augmentation for depression. We are interested in knowing if this is true for our sample, which was first reported to be a negative trial for celecoxib after 6 weeks (Baune et al., 2021), but showed relevant clinical effects after 35 weeks (Sampson et al., 2025).

We perform an analysis looking at treatment efficacy after 6 weeks, stratified by age. We test our central hypothesis that age affects immune-modulation and treatment efficacy in a randomized controlled sample treated with the anti-inflammatory agent celecoxib by modeling the interaction between age and treatment groups and how this interaction relates to treatment response. We aim to identify more homogeneous subgroups within the sample to better predict which patients could benefit most from an anti-inflammatory add-on therapy for depression.

For a more in-depth understanding of the mechanisms that could be involved in these processes, e.g., immunosenescence, we extended our analyses towards DNA-methylation-based cell type composition at baseline in a second step. These six well-known cell types have already been discussed in the context of depression and treatment response (Walther et al., 2022; Van Assche et al., 2025). Hence, we aim to explore the role of these six cell types in our newly defined age-dependent subgroups to better understand the role of immune-parameters, such as these cell types, in the context of age-dependent response to immunomodulatory treatment, and to investigate their predictive value as additional biomarkers.

## 2 Methods

### 2.1 Sample description

Our sample consisted of 119 individuals with Major Depressive Disorder (MDD) at baseline. Diagnoses were validated using DSM-IV-TR (American Psychiatric Association, 1998). The recruitment design was enriched for inflammation: patients were recruited and either included in the latent inflammation (hsCRP > 3 mg/L) group or the group without inflammation (hsCRP < 3 mg/L). Based on previous research, hsCRP is seen as a reliable and accessible biomarker reflecting low-grade information and peripheral inflammation in the context of depression (Osimo et al., 2019; van Dooren et al., 2016; Suneson et al., 2023), also linked to the structural changes in the brain (Opel et al., 2019). This stratified recruitment strategy is not of further interest to this manuscript. Nonetheless, hsCRP as a continuous variable was included as a covariate in the analyses to account for unwanted effects that might have arisen following the recruitment strategy. Inclusion criteria were a diagnosis of MDD with MADRS score ≥26, aged 18–75 years. Patients had to be outpatients in a psychiatric setting at screening and baseline visits and have at least one prior episode of depression, validated by previous treatment. The current major depressive episode (MDE) had to be confirmed by the MINI and had to have a duration of at least three months.

Patients were excluded when there was another co-morbid psychiatric disorder in the focus of clinical concern, or they had a current alcohol and/or substance use disorder. Patients with a primary inflammatory or immune-related disorder, a recent systemic infection, or taking immunosuppressant medications were excluded, as well as patients with a neurodegenerative disorder or a history of neurological disorder. Also, patients with an increased risk for side-effects regarding the study medication were excluded, i.e., a past peptic ulcer disease or history of gastrointestinal (GI) bleeding, an unstable coronary artery, or cardiovascular disease, or any previous hypersensitivity to vortioxetine or celecoxib, or the use of concomitant medications able to affect cognitive function or to induce drug–drug interaction. Further exclusion criteria include renal impairment or any other physical, cognitive, reading, learning, or language impairment.

An in-depth description of the study design can be found elsewhere (Fourrier et al., 2018).

As part of this trial, all individuals were treated with the antidepressant vortioxetine, but patients were randomised for an augmentation treatment with celecoxib (10 mg) or placebo. Participants were treated with celecoxib or placebo add-on therapy for the duration of 6 weeks.

Blood was collected at multiple time points. For this manuscript, we focus on baseline molecular markers only.

Following quality control (QC) for the molecular markers, 113 individuals were included in the analyses (*M*_age_ = 44, 56% women), resulting in 55 individuals with treatment augmentation with celecoxib and 58 individuals receiving placebo. A detailed description of sample characteristics is shown in Table 1.

**Table 1:** Descriptive statistics of samples after randomization.

	Vortioxetine + Celecoxib	Vortioxetine + Placebo	Statistical comparison of both groups
*N*	55	58	
% men	49%	40%	*p* = 0.35
Mean age (years); SD	44; 13.31	45; 15.18	*F*(1,111) = 0.071; *p* = 0.79
Mean MADRS at baseline; SD	29.13; 6.89	26.43; 5.83	*F*(1,111) = 5.07; *p* = 0.026
Mean BMI; SD	29.58; 6.84	29.93; 7.46	*F*(1,111) = 0.034; *p* = 0.85
Mean Years of education; SD	14.2; 2.3	14.0; 2.2	*F*(1,111) = 0.27; *p* = 0.61
Median hsCRP	0.9	1.9	chi^2^ = 0.81; *p* = 0.36

Groups of patients treated with Vortioxetine and Celecoxib or Vortioxetine and placebo are comparable for demographic covariates of interest. A significant difference is only seen for MADRS at baseline. MADRS at baseline and all other covariates are included in the model.

Depression severity was measured using the Montgomery-Asberg Depression Rating Scale (MADRS) at baseline and after the treatment period with celecoxib, i.e., 6 weeks. The mean MADRS at baseline was 27.7 (*SD* = 6.5), reflecting moderate to severe depression for most participants. In the context of the cohort, longitudinal phenotypic data are available as well, including MADRS after a 6-week interval, following treatment (*M* = 20.2; *SD* = 10.8).

Data were collected in Adelaide, Australia, between 2017 and 2020 (Fourrier et al., 2018). The study and data collection have been approved by the human research ethics committees of the Royal Adelaide Hospital and the University of Adelaide (reference number R20170320 HREC/17/RAH/111), and pre-registered on the Australian New Zealand Clinical Trials Registry (ACTRN12617000527369; https://www.anzctr.org.au/Trial/Registration/TrialReview.aspx?ACTRN=12617000527369p).

### 2.2 Assessment of molecular biomarkers

All 119 participants provided whole blood for molecular analyses. DNA was isolated from whole blood samples using standard procedures (QIAamp DNA Blood Midi-Kit, Qiagen, Hilden, Germany), followed by purification (Amicon 0.5 ml 3K; Merck/Millipore, Darmstadt, Germany) and pipetting on 96-well plates for chip-based analyses. Samples were randomized on plates and chips based on patients’ sex, age, and treatment. The Illumina Infinium MethylationEPIC 850k BeadChip was used for DNA-methylation analysis. Bisulfite conversion and handling of the DNA methylation chips were performed in the Life&Brain Institute, Bonn (Zillich et al., 2022). Following analysis on HiScan array scanning systems (Illumina, San Diego, CA), data were transferred as.idat files.

The ‘RnBeads’ pipeline (Package RnBeads 2.0 (Assenov et al., 2014; RnBeads 2), in R (version 4.3.1)) was used for processing and QC measures of the epigenome-wide DNA methylation data. Following QC procedures, 113 participants were included for the baseline measurement (for details on QC, see supplementary materials, Table S1).

The available epigenome-wide DNA-methylation data were used to estimate the immunological cell type composition using the Houseman method (Houseman et al., 2014) embedded in the RnBeads commands (rnb.execute.ct.estimation(), settings: test.max.markers = 10000, top.markers = 500). As a validated reference, we used the dataset GSE110554, published by Salas et al. (2018). We estimated 6 cell types of interest to depression, based on prior research (Walther et al., 2022; Van Assche et al., 2025) (neutrophils, natural killer cells (NK), B cells, CD4^+^ T cells, CD8^+^ T cells, monocytes). This method returns an estimated proportion for each of the cell types, for each of the individuals, based on the distinct methylomic signature for each cell type.

### 2.3 Statistical analyses

All analyses were performed with linear regression models and ANCOVA in R with MADRS_6W_ as the outcome of interest. Our primary hypothesis: the interaction between age and treatment group, was tested in the full sample, corrected for sex, hsCRP, BMI, years of education, and depression severity at baseline. We tested one central hypothesis: the interaction of age and treatment condition for treatment response. Hence, we decided not to perform any additional correction for multiple testing. The analyses focusing on cell type composition are secondary analyses to better understand the immunological properties of the relationship tested in our hypothesis.

All secondary analyses were corrected for the same covariates, too, as were analyses looking into cell type composition stratified by age. For the analyses focusing on cell types, the intervention group was not included in the models as an additional variable. Correlations per group were calculated using Pearson's correlation coefficient instead.

The intersection was calculated using the coefficients of both regression lines (m1 and m2, respectively) according to the following formula: x = −(intercept[m1]-intercept[m2])/(slope[m1]-slope[m2]), y = slope[m1]∗x + intercept[m1].

Descriptive statistics on demographic variables were performed with ANOVA and Fisher's exact test, and the non-parametric Kruskal-Wallis rank sum test for hsCRP.

## 3 Results

### 3.1 Sample description: celecoxib vs. placebo

A description of both groups and their comparability regarding covariates of interest is presented in Table 1.

### 3.2 Age, treatment group, and treatment response

**Fig. 1:**
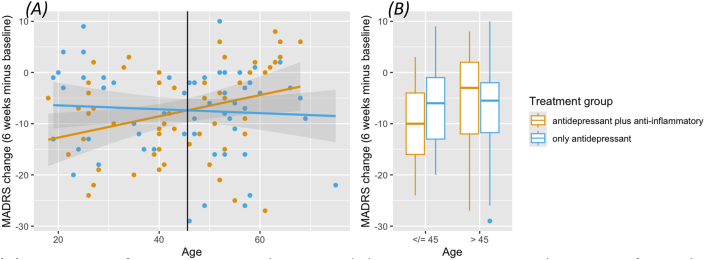
Treatment group-by-age Interaction for change in MADRS. (A) Interaction of treatment group-by-age and change in MADRS over the course of 6 weeks. Black line: intersection at 45.62 years. (B) Individuals stratified by age: 45 years or younger versus older than 45 years.

Our analysis showed a statistically significant interaction (Fig. 1A) between age and treatment condition, with the reduction in depression severity over the course of the trial as outcome (beta = −0.24; *p* = 0.045). Both main effects were significant too in the model (intervention: beta = 10.88; *p* = 0.048, age: beta = 0.23; *p* = 0.012). Sex, years of education, and hsCRP were no statistically significant contributors (*p* > 0.48). BMI was a marginally significant contributor in the model (beta = −0.25; *p* = 0.059). MADRS at baseline was a highly significant predictor for MADRS after six weeks of treatment (beta = 0.97; *p* < 0.001). The full model tested is included in the supplementary data.

Following the significant interaction between age and treatment condition, we identified the intersection, which was at 45.62 years (Fig. 1A). Hence, we stratified the sample for further analyses with 45 years as a cut-off (Fig. 1B): we grouped individuals who are 45 years old or younger and individuals older than 45 years (starting from 46 years). Details are summarized in Table 2.

**Table 2:** Descriptive statistics of both age groups.

	45 years and younger	Older than 45 years	
*N*	53	60	
% men	47%	42%	*p* = 0.57
Mean age (years); SD	32; 8.35	56; 6.49	*F*(1,111) = 317.8; *p <* 0.001
Mean MADRS at baseline; SD	26.96; 6.89	28.43; 6.65	*F*(1,111) = 0.63; *p* = 0.43
Mean BMI; SD	28.91; 6.25	30.60; 6.40	*F*(1,111) = 0.54; *p* = 0.46
Mean Years of education; SD	14.28; 2.22	13.87; 2.22	*F*(1,111) = 0.009; *p* = 0.93
Median hsCRP	1.0	1.8	chi^2^ = 0.66; *p* = 0.42

Apart from age, both groups are very comparable regarding potential confounding factors, especially MADRS at baseline.

Only for individuals treated with celecoxib, age played a significant role in treatment response (*F*(1,52) = 5.74; p = 0.020). Individuals aged 45 or younger showed a reduction of MADRS of 9.6 points over the course of 6 weeks. Individuals older than 45 years treated with celecoxib showed a reduction of 5.5 points (Fig. 1B). For individuals treated with vortioxetine and placebo, i.e., vortioxetine monotherapy, there were no age-related differences (*F*(1,55) = 0.02; p = 0.88), with a mean reduction in MADRS of 6.8 points for younger individuals and 7.8 for individuals older than 45 years (Fig. 1B). Overall, treatment response was significantly different between treatment groups (celecoxib vs. placebo) only for individuals 45 years or younger (beta = 4.18; *p* = 0.049)

### 3.3 Age-stratified analyses of estimated cell types

None of the cell types showed statistically significant differences between both age groups, except for a statistical trend for CD8^+^ T cells (*F*(1,111) = 2.92; *p* = 0.090; see supplementary data).

Based on the significant interaction between treatment and age in our sample, we investigated the role of baseline cell type estimates and their effect depending on age group and treatment condition.

**Fig. 2:**
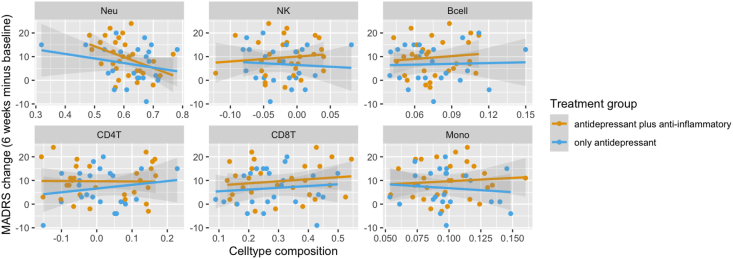
Cell type composition at baseline and reduction in MADRS over time for individuals 45 years and younger Depiction of the associations between cell type composition and change in MADRS over the course of 6 weeks. Regression lines indicate the treatment group. Panes from top left to bottom right: Neutrophiles, Natural Killer cells, B-cells, CD4-T cells, CD8-T cells, Monocytes.

*Individuals aged 45 years and younger.* For individuals 45 years and younger, our data suggested that neutrophils at baseline were related to response to treatment (beta = 25.87; *p* = 0.051). Only for individuals treated with celecoxib, there was a statistically significant correlation between neutrophils at baseline and MADRS reduction after 6 weeks (*r*(27) = −0.41, *p* = 0.028). There was no such correlation for individuals having received a placebo (*r*(22) = −0.24, *p* = 0.26). Other cell types did not show an association with a change in MADRS over the course of treatment and were not further investigated (see Fig. 2).

**Fig. 3:**
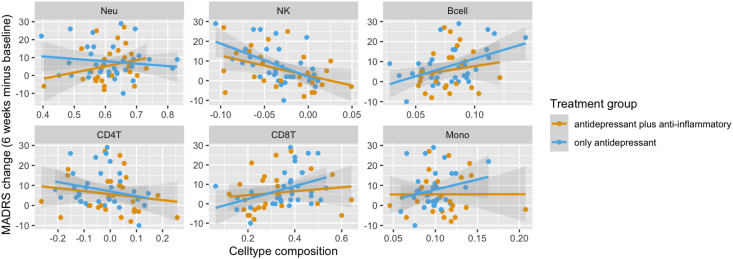
Cell type composition at baseline and reduction in MADRS over time for individuals older than 45 years Depiction of the associations between cell type composition and change in MADRS over the course of 6 weeks. Regression lines indicate the treatment group. Panes from top left to bottom right: Neutrophiles, Natural Killer cells, B-cells, CD4-T cells, CD8-T cells, Monocytes.

*Individuals older than 45 years.* For individuals older than 45 years, the proportions of natural killer (NK) cells (beta = 129.39; *p* < 0.001) and B-cells (beta = −146.29; *p* = 0.014) at baseline were significantly associated with MADRS after six weeks of treatment (see Fig. 3). The proportions of CD8^+^ T-cells at baseline suggested a potential association with a reduction in MADRS over the course of treatment (beta = −20.23; *p* = 0.055).

For all three cell types, correlations between baseline cell type composition and reduction in MADRS were strongest for the group receiving placebo (see Fig. 3). There was a negative correlation between NK proportions and MADRS reduction for both groups (celecoxib: *r*(24) = −0.41, *p* = 0.035, placebo: *r*(32) = −0.52, *p* = 0.0016). For B-cells and CD8^+^ T-cells, there was a positive correlation for individuals treated with placebo (B-cells: placebo: *r*(32) = 0.47, *p* = 0.0056; celecoxib: *r*(24) = 0.16, *p* = 0.43; CD8^+^ T-cells: placebo: *r*(32) = 0.39, *p* = 0.024; celecoxib: *r*(24) = 0.13, *p* = 0.38).

## 4 Discussion

In line with our hypothesis, our data show that age is a relevant actor in the relationship between augmentation with an immunomodulatory agent, such as celecoxib, and treatment response. Our data-driven approach marked 45 years as a relevant cut-off in our sample.

We could show that individuals 45 years or younger showed a stronger response to the augmentation with celecoxib, whereas this effect was absent for individuals older than 45 years. For younger individuals, treatment response was significantly better when receiving celecoxib as an augmenting agent, compared to placebo. This age-dependent effect on treatment response was not seen in the individuals receiving vortioxetine augmented with placebo. For individuals older than 45 years, there was also no difference in response between the groups treated with celecoxib augmentation or placebo.

Individuals older than 45 years with lower NK proportions, higher B-cell proportions, and higher CD8^+^ T cell proportions profited most from treatment, most pronouncedly treatment *without* immunomodulatory augmentation. This observation paradoxically suggests an immune-modulated mechanism after all. For individuals 45 years and younger, a lower neutrophil count at baseline proved to be particularly beneficial for treatment response with celecoxib as an augmenting agent.

Our exploratory analyses with estimated cell type compositions supported the altered immunological profile and involvement with age. These analyses suggest the role of cell type in treatment response varies with age, too. Our data show that cell type composition at baseline is a better predictor for treatment response for elderly individuals, particularly individuals not receiving an augmentation treatment with celecoxib. This is not due to the cell types per se, as there were no significant differences between the two age groups for the cell types.

In line with our data, the existing literature, e.g., the meta-analysis by Wang et al. (2022) showed higher effect sizes of celecoxib for individuals with drug-naive depression and mild to moderate depression. Both aspects are typically linked to a shorter disease history and a potential younger age. The studies reporting the higher effect sizes report trials with mean ages below 45 years (Majd et al., 2015; Akhondzadeh et al., 2009). Also, one of the earlier meta-analyses documenting additional benefit for celecoxib in treating depression presents samples mostly below 45 years of age (Na et al., 2014), and the pioneering study of Müller et al. report a mean age of 44.5 years (Müller et al., 2006). Another study comparing celecoxib and diclofenac for augmentation of the antidepressant therapy in breast cancer patients reports an overall suboptimal effect of the intervention, with no one reaching remission after a 6-week trial, though effects were better for celecoxib than diclofenac. The reported mean age of the sample was 58 years.

Our data-driven approach marked 45 years as a relevant cut-off in our sample. The age span between 40 and 60, i.e., “middle-age”, is increasingly of interest to scientists, as it appears to be a turning point with impact on neurodegenerative and other processes typical for old(er) age (Dohm-Hansen et al., 2024). Some physiological processes are known to change around that age; menopause is one example that also affects immunological processes. However, the identified age groups were well-balanced regarding sex distribution. Nonetheless, there is increasing evidence that cerebral processes of neurodegeneration – often also linked to immunological processes - have a long subclinical prodromal phase, e.g., Parkinson's disease (Postuma and Berg, 2016). Further research will have to help understand the meaning of these results and the role and impact of physiological changes on the middle-aged brain.

As aging is not a linear process, acceleration and deceleration of age-related changes are to be assumed. Shen et al. (2024) could show that the ages of 44 and 60 years mark two time-points most prone to physiological age-acceleration, based on multi-omics data, which is in line with our cut-off.

For now, it is hard to speculate to what extent these data can be extrapolated to other anti-inflammatory agents, as their modes of action are very different (e.g., minocycline, an antibiotic, vs. celecoxib, an NSAID). Though overall trials have been mostly inconclusive, there seems to be increasing evidence that celecoxib is superior as an anti-inflammatory augmentation for depression, compared to other immune-modulatory agents (Simon et al., 2023b).

Also, pharmacokinetics and pharmacodynamics are processes that change with age. An altered metabolism of celecoxib may contribute to the observed effects.

An important limitation in this context is that we have no measure of chronicity to distinguish between age or a long history of depression, spanning years. Relying on the Maudsley's staging method, a history of electroconvulsive therapy (ECT), multiple medications, or treatment augmentation are some indicators of chronicity (Fekadu et al., 2018). As far as we can reconstruct chronicity in our sample with the information available, the three individuals who received ECT are among the patients older than 45 years, and so are 12 out of 15 individuals who reported more than one antidepressant, an antipsychotic, or mood stabilizer, which can be interpreted as augmentation, and an indirect indicator of chronicity. Hence, the lack of response to an immunomodulatory agent and the altered cell-type profile can also be a consequence of a longer disease history and a more chronic course of depression. In this light, adding celecoxib might particularly benefit individuals with a first or second episode of depression. Overall, however, older individuals have been known to respond worse to antidepressant therapies than younger individuals (Ossola et al., 2025). Our data add to the understanding of this general observation, as our results indicate that age-related immunological changes and immunosenescence may be underlying factors contributing to worse antidepressant treatment response in older individuals.

Other limitations include that a larger sample size might have been more informative and more convincing in transferring the message. However, despite the sample size, we could demonstrate the role of age through multiple secondary analyses that supported our primary hypothesis. Also, that we only looked at six DNA-methylation estimated cell types can be seen as a limitation: the immunological aspects of depression and age are much more complex than estimates of cell type compositions can show.

As more trials with anti-inflammatory agents for depression are being set up, we advise colleagues to perform well-argued stratified analyses. Sex-based stratification is becoming a standard analytical approach. For processes related to immune response, an age-by-treatment interaction model or age-based stratification should be considered, too. Our data suggest that age, and the immunological processes it inherently encompasses, e.g., immunosenescence, can be a relevant factor contributing to the heterogeneity of the treatment outcome. Applying evidence-based stratification strategies can bring the field forward, as more homogeneous patient groups will enable more conclusive results and facilitate the transfer into clinical practice.

## 5 Conclusion

Overall, our results suggest that immunological profiles in depression and patients’ response to immunomodulatory treatment may be age- or chronicity-dependent. These results can inform the new studies that are being set up and can serve as an independent replication sample. Immunosenescence is an emerging concept highlighting the impact of aging on the immune system. Our research opens a small window and offers a perspective on the role immunosenescence can play in treatment efficacy and personalized treatment choices based on the aging of the immune system and its impact on treatment response. If these results are confirmed, we are one step closer to tackling heterogeneity and establishing a patient profile that may benefit particularly from adding celecoxib or other immunomodulatory augmentation strategies to the antidepressant therapy.

## Funding sources

Bernhard T. Baune received support from Psych-STRATA, a project funded by the European Union's Horizon Europe research and innovation programme under Grant Agreement No. 101057454.

## Declaration of competing interest

The authors declare that they have no known competing financial interests or personal relationships that could have appeared to influence the work reported in this paper.
