# Supplementary material for: Response to immune-based augmentation treatment for depression: a potential role of immunosenescence
Source: Neurosci Appl. 2026 Feb 6;5:106986. doi: 10.1016/j.nsa.2026.106986 (PMC12933617; doi:10.1016/j.nsa.2026.106986)
Supplement: Multimedia component 1 [file mmc1.docx]

**Supplementary Data: Response to immune-based augmentation treatment for depression: a potential role of immunosenescence**

**QC of DNA methylation**

**Phenotype:**

Overall sample at baseline: *N*=119

DNA methylation available (Illumina EPIC-idat): *N*=116

Following QC, three additional samples were removed: *N*=113

- Technical issues and issues and bisulfite conversion controls: 3 samples

**Epigenomic QC**

Settings for preprocessing:

Normalisation method: wm.dasen normalisation (WateRmelon)

Greedycut: detection p-value cut-off 0.05

Removal of: SNP-related CGs, Sex-related CGs, cross-reactive probes

*Table S1: Epigenomic QC*

|  | **Probes at start** | **Samples at start** |
| --- | --- | --- |
| **Total at start preprocessing** | **866.895** | **113** |

| **Step (Filtering I)** | **Probes removed** | **Samples removed** |
| --- | --- | --- |
| Removal of SNP-enriched Probes | 139.721 | *0* |
| Removal of Cross-reactive Probes | 34.264 | *0* |
| Greedycut | 8.587 | *0* |
|  |  |  |
| *Total removed* | *182.572* | *0* |

|  | **Probes retained** | **Samples retained** |
| --- | --- | --- |
| **Total retained** | 684.323 | **113** |

| **Step (Filtering II)** | **Probes removed** | **Samples removed** |
| --- | --- | --- |
| Context-specific Probes | 1.052 | *0* |
| Removal of Probes on Sex Chromosomes | 16.223 | *0* |
| Probes with Missing values > 90% | 153 | *0* |
|  |  |  |
| *Total removed* | *17.428* | *0* |

|  | **Probes retained** | **Samples retained** |
| --- | --- | --- |
| **Total retained** | 666.895 | **113** |

**Main hypothesis**

MADRS_6W_ ~ age * Intervention + gender + hsCRP + MADRS_BL_ + BMI + education

*Residuals: Min 1Q Median 3Q Max*

-22.36 -5.86 1.72 5.78 16.49

*Coefficients: Estimate Std.Error t-value p-value*

(Intercept) -7.20 8.35 -0.86 0.39

**Age 0.23 0.089 2.54 0.012**

**Intervention (AD)* 10.88 5.44 2.00 0.048**

Gender (M)* 1.19 1.69 0.71 0.48

hsCRP 0.056 0.22 0.26 0.80

MADRS_BL_ 0.97 0.13 7.40 3.65x10^-11^

BMI -0.25 0.13 -1.91 0.059

Education -0.22 0.37 -0.59 0.56

**Age x Intervention (AD) -0.24 0.12 -2.03 0.045**

Qualities of the model:

Adj. *R^2^*= 0.36, *F*(8,104) = 8.85, *p*= 3.59x10^-9^; Residual SE: 8.66 on 104 degrees of freedom

**AD= antidepressant only; M = male*

**Stratified analyses**

**Individuals 45 years and younger**

MADRS_6W_ ~ Intervention + gender + hsCRP + MADRS_BL_ + BMI + education

*Residuals: Min 1Q Median 3Q Max*

-16.85 -4.09 0.13 3.68 14.08

*Coefficients: Estimate Std.Error t-value p-value*

(Intercept) -23.60 10.19 -2.32 0.025

**Intervention (AD) 4.18 2.07 2.02 0.049**

Gender (M) 6.12 2.11 2.90 0.0057

hsCRP 0.15 0.26 0.60 0.55

MADRS_BL_ 1.29 0.18 7.14 5.6x10^-9^

BMI -0.25 0.14 -1.81 0.077

Education 0.65 0.45 1.43 0.16

Qualities of the model:

Adj. *R^2^*= 0.52, *F*(6,46) = 10.26, *p*= 3.41x10^-7^; Residual *SE*: 7.07 on 46 degrees of freedom

**Individuals older than 45 years**

MADRS_6W_ ~ Intervention + gender + hsCRP + MADRS_BL_ + BMI + education

*Residuals: Min 1Q Median 3Q Max*

-21.95 -5.98 3.56 5.69 16.46

*Coefficients: Estimate Std.Error t-value p-value*

(Intercept) 12.46 11.76 1.06 0.29

**Intervention (AD)** -3.11 2.72 -1.14 0.26

Gender (M) -0.92 2.71 -0.34 0.74

hsCRP -0.24 0.35 -0.69 0.50

MADRS_BL_ 0.87 0.20 4.34 6.39x10^-5^

BMI -0.11 0.23 -0.49 0.63

Education -0.66 0.59 -1.12 0.27

Qualities of the model:

Adj. *R^2^*= 0.25, *F*(6,53) = 4.21, *p*= 0.0015; Residual *SE*: 9.78 on 53 degrees of freedom

**Models with cell types**

*Df Sum Sq. Mean Sq. F-value p-value*

*Neutrophiles ~ age Group*

Age Group 1 0.000 0.00 0.004 0.95

Residuals 111 0.77 0.0069

*Natural Killer cells ~ age Group*

Age Group 1 0.0033 0.0033 2.45 0.12

Residuals 111 0.15 0.0013

*Monocytes ~ age Group*

Age Group 1 0.00046 0.00046 0.73 0.39

Residuals 111 0.070 0.00063

*B-cells ~ age Group*

Age Group 1 0.00004 0.000040 0.079 0.78

Residuals 111 0.056 0.00051

*CD4+ T-cells ~ age Group*

Age Group 1 0.021 0.021 2.05 0.16

Residuals 111 1.16 0.010

*CD8+ T-cells ~ age Group*

Age Group 1 0.044 0.044 2.92 0.090

Residuals 111 1.66 0.015

**Individuals 45 years and younger**

***MADRS_6W_ ~ NK + hsCRP + gender + BMI + education + MADRS_BL_***

*Residuals: Min 1Q Median 3Q Max*

-18.39 -3.52 -0.44 3.37 14.01

*Coefficients: Estimate Std.Error t-value p-value*

(Intercept) -17.76 10.20 -1.74 0.089

Natural Killer cells -12.69 27.38 -0.46 0.65

hsCRP 0.18 0.27 0.67 0.51

Gender (M) 5.38 2.16 2.49 0.017

BMI -0.27 0.14 -1.9 0.061

Education 0.60 0.47 1.26 0.21

MADRS_BL_ 1.20 0.18 6.50 5.11x10^-8^

Adj. *R^2^*= 0.48, *F*(6,46) = 8.88, *p*= 1.92x10^-6^; Residual *SE*: 7.36 on 46 degrees of freedom

***MADRS_6W_ ~ Neu + hsCRP + gender + BMI + education + MADRS_BL_***

*Residuals: Min 1Q Median 3Q Max*

-18.49 -3.77 -0.37 4.46 13.70

*Coefficients: Estimate Std.Error t-value p-value*

(Intercept) -35.99 13.56 -2.65 0.011

**Neutrophiles 25.87 12.92 2.003 0.051**

hsCRP 0.018 0.27 0.065 0.95

Gender (M) 4.72 2.09 2.26 0.029

BMI -0.19 0.14 -1.35 0.18

Education 0.59 0.45 1.30 0.20

MADRS_BL_ 1.24 0.18 7.10 6.51x10^-9^

Adj. *R^2^*= 0.52, *F*(6,46) = 10.24, *p*= 3.52x10^-7^; Residual *SE*: 7.07 on 46 degrees of freedom

Pearson's correlation: change in MADRS and Neutrophiles

**Celecoxib: *t*(27)= -2.33; *p*-value = 0.028; correlation: -0.41 (CI: -0.67-0.050)**

Placebo: *t*(22)= -1.15; *p*-value = 0.26; correlation: -0.24 (CI: -0.59-0.18)

***MADRS_6W_ ~ Mono + hsCRP + gender + BMI + education + MADRS_BL_***

*Residuals: Min 1Q Median 3Q Max*

-17.97 -3.18 -0.77 3.49 14.90

*Coefficients: Estimate Std.Error t-value p-value*

(Intercept) -15.60 10.51 -1.48 0.14

Monocytes -18.94 46.55 -0.41 0.69

hsCRP 0.14 0.28 0.50 0.62

Gender (M) 5.54 2.23 2.48 0.017

BMI -0.26 0.14 -1.82 0.076

Education 0.59 0.47 1.25 0.22

MADRS_BL_ 1.20 0.18 6.53 4.6x10^-8^

Adj. *R^2^*= 0.48, *F*(6,46) = 8.86, *p*= 1.97x10^-6^; Residual *SE*: 7.36 on 46 degrees of freedom

***MADRS_6W_ ~ Bcell + hsCRP + gender + BMI + education + MADRS_BL_***

*Residuals: Min 1Q Median 3Q Max*

-17.79 -3.17 -0.86 3.86 14.14

*Coefficients: Estimate Std.Error t-value p-value*

(Intercept) -16.07 10.76 -1.49 0.14

B-cells -9.69 46.45 -0.21 0.84

hsCRP 0.17 0.27 0.63 0.53

Gender (M) 5.26 2.17 2.43 0.019

BMI -0.27 0.14 -1.90 0.064

Education 0.61 0.48 1.28 0.21

MADRS_BL_ 1.17 0.19 6.27 1.13x10^-7^

Adj. *R^2^*= 0.47, *F*(6,46) = 7.37, *p*= 2.83x10^-6^; Residual *SE*: 7.37 on 46 degrees of freedom

***MADRS_6W_ ~ CD4T + hsCRP + gender + BMI + education + MADRS_BL_***

*Residuals: Min 1Q Median 3Q Max*

-17.93 -2.86 -0.80 3.71 14.15

*Coefficients: Estimate Std.Error t-value p-value*

(Intercept) -16.83 10.12 -1.66 0.10

CD4+ T-cells 0.44 11.96 0.037 0.97

hsCRP 0.17 0.27 0.64 0.53

Gender (M) 5.31 2.19 2.43 0.019

BMI -0.27 0.14 -1.92 0.061

Education 0.60 0.47 1.26 0.21

MADRS_BL_ 1.18 0.19 6.15 1.72x10^-7^

Adj. *R^2^*= 0.47, *F*(6,46) = 8.80, *p*= 2.12x10^-6^; Residual *SE*: 7.37 on 46 degrees of freedom

***MADRS_6W_ ~ CD8T + hsCRP + gender + BMI + education + MADRS_BL_***

*Residuals: Min 1Q Median 3Q Max*

-16.6 -3.47 -1.39 4.07 15.42

*Coefficients: Estimate Std.Error t-value p-value*

(Intercept) -13.52 10.51 -1.29 0.20

CD8+ T-cells -9.21 9.32 -0.99 0.33

hsCRP 0.11 0.28 0.40 0.69

Gender (M) 5.23 2.14 2.44 0.018

BMI -0.26 0.14 -1.85 0.071

Education 0.63 0.47 1.34 0.19

MADRS_BL_ 1.14 0.18 6.18 1.54x10^-7^

Adj. *R^2^*= 0.48, *F*(6,46) = 9.15, *p*= 1.36x10^-6^; Residual *SE*: 7.30 on 46 degrees of freedom

**Individuals older than 45 years**

***MADRS_6W_ ~ NK + hsCRP + gender + BMI + education + MADRS_BL_***

*Residuals: Min 1Q Median 3Q Max*

-18.80 -4.80 1.26 5.69 22.10

*Coefficients: Estimate Std.Error t-value p-value*

(Intercept) 7.83 10.44 0.75 0.46

**Natural Killer cells 129.39 35.34 3.66 0.00058**

hsCRP 0.16 0.31 0.51 0.61

Gender (M) -0.49 2.45 -0.20 0.84

BMI -0.21 0.20 -1.06 0.30

Education -0.16 0.54 -0.30 0.77

MADRS_BL_ 0.92 0.18 5.20 3.27x10^-6^

Adj. *R^2^*= 0.38, *F*(6,53) = 7.12, *p*= 1.34x10^-5^; Residual *SE*: 8.84 on 53 degrees of freedom

Pearson's correlation: change in MADRS and Natural Killer cells

**Celecoxib: *t*(24)= -2.23; *p*-value = 0.035; correlation: -0.41 (CI: -0.69-0.033)**

**Placebo: *t*(32)= -3.45; *p*-value = 0.0016; correlation: -0.52 (CI: -0.73-0.22)**

***MADRS_6W_ ~ Neu + hsCRP + gender + BMI + education + MADRS_BL_***

*Residuals: Min 1Q Median 3Q Max*

-23.69 -5.53 2.76 6.50 16.92

*Coefficients: Estimate Std.Error t-value p-value*

(Intercept) 7.04 13.94 0.51 0.62

Neutrophiles 6.00 16.40 0.37 0.72

hsCRP -0.16 0.35 -0.46 0.65

Gender (M) -0.72 2.74 -0.26 0.80

BMI -0.17 0.22 -0.75 0.46

Education -0.63 0.60 -1.05 0.30

MADRS_BL_ 0.90 0.20 4.51 3.67x10^-5^

Adj. *R^2^*= 0.23, *F*(6,53) = 3.93, *p*= 0.0025; Residual *SE*: 9.88 on 53 degrees of freedom

***MADRS_6W_ ~ Mono + hsCRP + gender + BMI + education + MADRS_BL_***

*Residuals: Min 1Q Median 3Q Max*

-23.49 -5.72 2.53 6.01 16.85

*Coefficients: Estimate Std.Error t-value p-value*

(Intercept) 13.14 13.09 1.00 0.32

Monocytes -30.87 55.94 -0.55 0.58

hsCRP -0.14 0.34 -0.43 0.67

Gender (M) -0.24 2.91 -0.082 0.94

BMI -0.18 0.22 -0.78 0.44

Education -0.55 0.60 -0.91 0.37

MADRS_BL_ 0.89 0.20 4.38 5.63x10^-5^

Adj. *R^2^*= 0.23, *F*(6,53) = 3.97, *p*= 0.0024; Residual *SE*: 9.87 on 53 degrees of freedom

***MADRS_6W_ ~ Bcell + hsCRP + gender + BMI + education + MADRS_BL_***

*Residuals: Min 1Q Median 3Q Max*

-21.91 -5.51 1.14 5.88 14.72

*Coefficients: Estimate Std.Error t-value p-value*

(Intercept) 16.03 11.28 1.42 0.16

**B-cells -146.29 57.23 -2.56 0.014**

hsCRP -0.19 0.32 -0.60 0.55

Gender (M) -0.70 2.58 -0.27 0.79

BMI -0.081 0.21 -0.38 0.71

Education -0.41 0.56 -0.73 0.47

MADRS_BL_ 0.92 0.19 4.91 9.12x10^-6^

Adj. *R^2^*= 0.31, *F*(6,53) = 5.47, *p*= 0.00018; Residual *SE*: 9.34 on 53 degrees of freedom

Pearson's correlation: change in MADRS and B-cells

Celecoxib: *t*(24)= 0.80; *p*-value = 0.43; correlation: 0.16 (CI: -0.24-0.52)

**Placebo: *t*(32)= 2.97; *p*-value = 0.0056; correlation: 0.47 (CI: 0.15-0.69)**

***MADRS_6W_ ~ CD4T + hsCRP + gender + BMI + education + MADRS_BL_***

*Residuals: Min 1Q Median 3Q Max*

-22.85 -6.09 2.66 5.88 17.37

*Coefficients: Estimate Std.Error t-value p-value*

(Intercept) 6.96 11.70 0.60 0.56

CD4+ T-cells 16.94 13.05 1.30 0.20

hsCRP -0.0012 0.35 -0.003 1.00

Gender (M) -0.162960 2.74 -0.060 0.95

BMI -0.16 0.22 -0.71 0.48

Education -0.46 0.59 -0.77 0.44

MADRS_BL_ 0.92 0.20 4.70 1.92x10^-5^

Adj. *R^2^*= 0.25, *F*(6,53) =4.31, *p* = 0.0013; Residual *SE*: 9.74 on 53 degrees of freedom

***MADRS_6W_ ~ CD4T + hsCRP + gender + BMI + education + MADRS_BL_***

*Residuals: Min 1Q Median 3Q Max*

-22.11 -6.59 2.48 6.61 19.76

*Coefficients: Estimate Std.Error t-value p-value*

(Intercept) 15.33 11.61 1.32 0.19

**CD8+ T-cells -20.23 10.30 -1.97 0.054**

hsCRP -0.036 0.33 -0.11 0.91

Gender (M) -0.049 2.67 -0.018 0.99

BMI -0.18 0.22 -0.83 0.41

Education -0.51 0.57 -0.89 0.38

MADRS_BL_ 0.91 0.19 4.75 1.61x10^-5^

Adj. *R^2^*= 0.28, *F*(6,53) =4.83, *p* = 0.00054; Residual *SE*: 9.55 on 53 degrees of freedom

Pearson's correlation: change in MADRS and CD8+ T-cells

Celecoxib: *t*(24)= 0.69; *p*-value = 0.50; correlation: 0.14 (CI: -0.26-0.50)

**Placebo: *t*(32)= 2.36; *p*-value = 0.024; correlation: 0.39 (CI:** 0.054-0.64**)**
